# Visual Search in the Real World: Color Vision Deficiency Affects Peripheral Guidance, but Leaves Foveal Verification Largely Unaffected

**DOI:** 10.3389/fnhum.2015.00680

**Published:** 2015-12-22

**Authors:** Günter Kugler, Bernard M. 't Hart, Stefan Kohlbecher, Klaus Bartl, Frank Schumann, Wolfgang Einhäuser, Erich Schneider

**Affiliations:** ^1^Institute of Clinical Neurosciences, University of MunichMunich, Germany; ^2^German Center for Vertigo and Balance Disorders, University of MunichMunich, Germany; ^3^Neurophysics, Philipps University MarburgMarburg, Germany; ^4^Centre for Vision Research, York UniversityToronto, ON, Canada; ^5^Laboratoire Psychologie de la Perception, Université Paris DescartesParis, France; ^6^Institute of Cognitive Science, University of OsnabrückOsnabrück, Germany; ^7^Institute of Physics, Chemnitz University of TechnologyChemnitz, Germany; ^8^Institute of Medical Technology, Brandenburg University of Technology Cottbus – SenftenbergSenftenberg, Germany

**Keywords:** color vision, color vision deficiency, visual search, visual perception, real world behavior, deuteranomaly, eye tracker, gaze behavior

## Abstract

**Background:** People with color vision deficiencies report numerous limitations in daily life, restricting, for example, their access to some professions. However, they use basic color terms systematically and in a similar manner as people with normal color vision. We hypothesize that a possible explanation for this discrepancy between color perception and behavioral consequences might be found in the gaze behavior of people with color vision deficiency.

**Methods:** A group of participants with color vision deficiencies and a control group performed several search tasks in a naturalistic setting on a lawn. All participants wore a mobile eye-tracking-driven camera with a high foveal image resolution (EyeSeeCam). Search performance as well as fixations of objects of different colors were examined.

**Results:** Search performance was similar in both groups in a color-unrelated search task as well as in a search for yellow targets. While searching for red targets, participants with color vision deficiencies exhibited a strongly degraded performance. This was closely matched by the number of fixations on red objects shown by the two groups. Importantly, once they fixated a target, participants with color vision deficiencies exhibited only few identification errors.

**Conclusions:** In contrast to controls, participants with color vision deficiencies are not able to enhance their search for red targets on a (green) lawn by an efficient guiding mechanism. The data indicate that the impaired guiding is the main influence on search performance, while foveal identification (verification) is largely unaffected by the color vision deficiency.

## Introduction

People with color vision deficiencies experience many limitations in various areas of daily life, e.g., in medical professions (Spalding et al., 2010), while identifying traffic signal colors (Atchison et al., 2003), or while reading figures in publications (Miall, 2007; Ross, 2007; Albrecht, 2010) (see Cole, 2004 for review). However, such a deficiency does not imply the inability to perceive any color. In that sense, the popular term “color blind” is misleading. For trichromats, it is possible to gain an impression of the residual color discrimination of dichromats by looking at pictures that are resampled in a two-dimensional color space (Viénot et al., 1995).

Color is known to have a strong influence on deployment of attention and eye movements, as a “guiding” characteristic in visual search (Wolfe et al., 1989; Wolfe, 2007), or as a “salient feature” captured in the saliency map (Koch and Ullman, 1985; Itti et al., 1998). This selection is crucial as the visual processing capacity is limited, and attention is necessary to confirm the presence of specific objects (Wolfe, 2010). By using color, scenes are segmented (Hansen and Gegenfurtner, 2009), and locations in the visual scene are selected for closer inspection.

Gaze behavior is often studied in the context of visual-search tasks. In visual search, targets and distractors are used to characterize the selection of items by their features. One ecologically valid example is foraging. Facilitated detection of ripe fruit might even have been involved as a driving factor in the evolution of primate trichromacy (Mollon, 1989), while most mammals are dichromats.

Real-world search remains a “key challenge” (Tatler, 2009), mainly due to the difficulties which the required experimental equipment is facing “in the wild.” So far, only few studies address real world search (Wolfe, 2010). We here, for the first time, examine gaze behavior of people with color vision deficiencies in an ecologically valid search task, which is similar to a search for fruits. Instead of berries, we distributed candies of various colors on a lawn. An open question is whether the search performance is related to the gaze behavior. We hypothesize that gaze behavior is affected by the color vision deficiency and that, consequently, search performance is worse, even for targets that can clearly be identified once fixated.

## Materials and methods

### Experimental set-up and task

**Figure 1:**
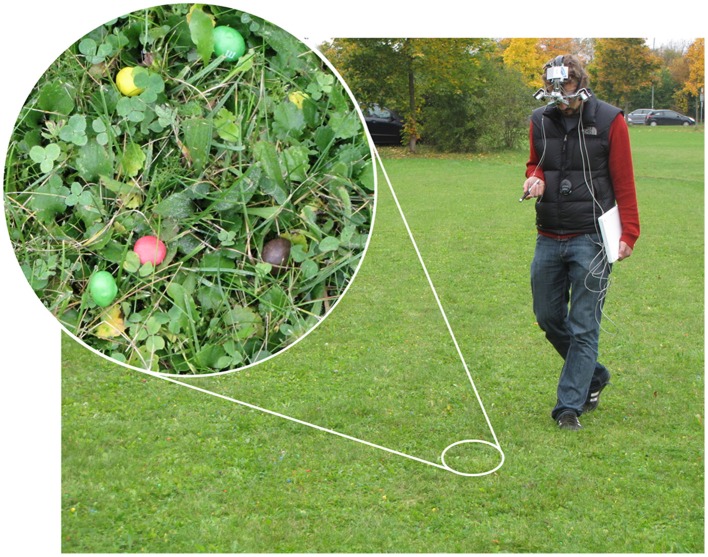
**Experimental setup: lawn with ~17,000 colored candies**. The participant is wearing a mobile eye-tracker, and carries a laser pointer and a laptop. In the top left a close-up shot of the lawn is shown.

**Figure 2:**
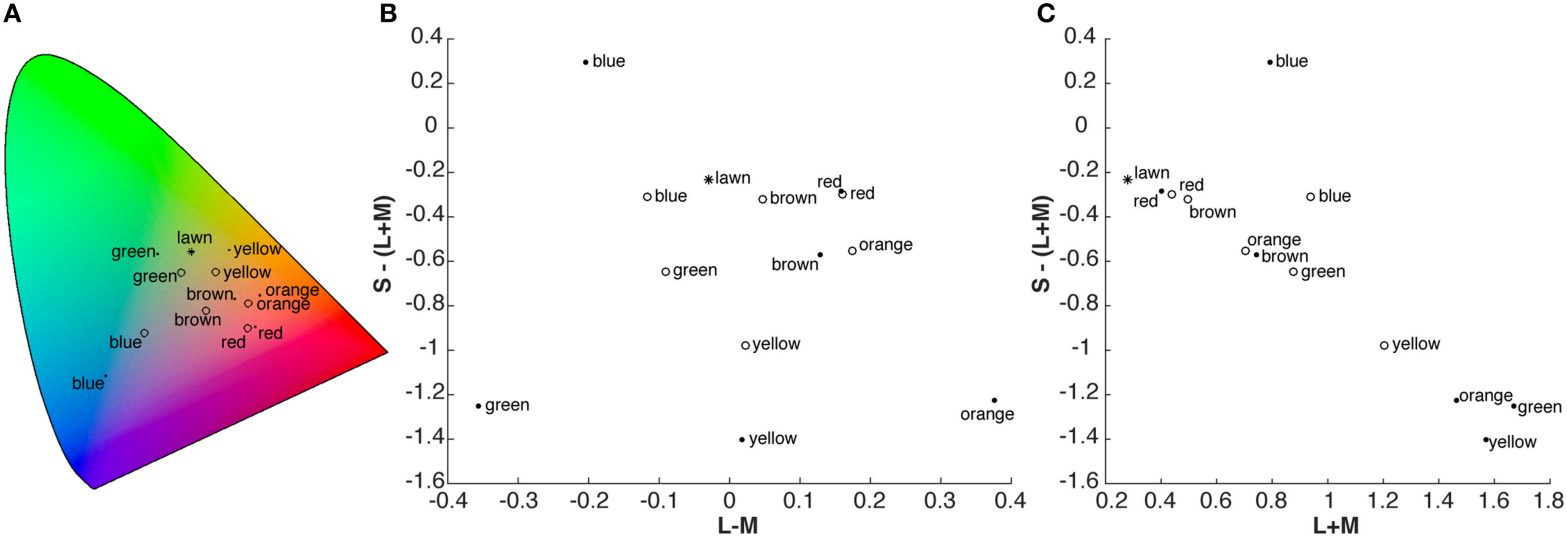
**(A)** Chromaticity coordinates of the targets (open symbols), distractors (filled symbols) and lawn (star) in CIE 1931 XYZ space. **(B,C)** Chromaticity coordinates in cone-contrast (DKL, Derrington et al., 1984), space. **(B)** Projection on L-M and S-(L+M) axes, **(C)** projection on L+M (proportional to luminance) and S-(L+M) axis. For participants with deficiencies along the L-M axis, only the information depicted in C is available, rendering the discrimination of red items from the lawn difficult.

We mimicked a search for fruit by distributing two types of candy on a lawn (Figure 1). The two different types of candies were distinguishable by shape, and thus categorized as either target or distractor. They were of the same six colors: red, green, yellow, blue, orange, and brown. Targets were also present in two additional colors: purple and pink. The chromaticity coordinates and luminance (xyY) of the targets, distractors, different samples of background lawn (Figure 2A), as well as of a reference white were measured with a Konica Minolta Chroma Meter CS 100 (Konica Minolta Sensing Americas Inc., NJ, USA). These values were converted to CIELAB space using the average measurement of the reference white as the adapting white-point (xyY = 0.308, 0.317, 844 cd/m^2^). The resulting CIELAB coordinates (L^*^,a^*^,b^*^) of the targets were: 53.3, 46.3, 18.8 (red); 64.7, 36.2, 36.0 (orange); 80.3, −0.4, 48.9 (yellow); 71.0, −21.6, 31.6 (green); 74.9, −11.1, −9.8 (blue); and 56.2, 15.1, 16.4 (brown). Distractors had CIELAB coordinates of: 51.1, 47.8, 21.0 (red); 86.6, 46.6, 57.9 (orange); 88.8, −5.5, 76.0 (yellow); 91.9, −58.2, 41.5 (green); 72.9, −11.2, −47.4 (blue); and 66.2, 26.3, 34.2 (brown). The average CIELAB coordinates of the background lawn were 43.3, −17.8, 31.2 (there was a range of chromaticities due to the texture of the lawn, with strongest variation in lightness (L^*^) and blue-yellow (b^*^) across samples). Differences between red items (targets and distractors) and the lawn were predominantly based on the contrast between L-cone and M-cone activation (Figure 2B), whereas S-cone and luminance provided little information to distinguish red items from the background (Figure 2C). In contrast, other items (except the brown target), in particular the yellow target and the yellow distractor, could be well discriminated from the lawn and from other items based on their luminance and S-cone activation (Figure 2C). DKL-space coordinates were calculated with matrix transformations according to the procedure described by Brainard (1996).

The target candies were round, flat and less frequent (3% of total objects, Smarties, Nestlé, Vevey, Switzerland), while the distractor candies were oval and less regular in shape (peanut M&Ms, Mars Inc. MacLean, VA, USA). Distractors also contained the letter “m” on their surface, which, however, is too small to be used as peripheral cue. In total, about 34,000 target and distractor candies were distributed on a meadow of about 1600 m^2^, resulting in a density of roughly 21 candies per square meter. To minimize effects of overnight bleaching (due to dew on the sugar-coated candies), we only used a subarea of the total lawn for each of four experimental days, and distributed candies at the respective subparts at the beginning of each experimental day. The assignment of participant to lawn was random, and each of the subareas was sufficiently large that boundaries were rarely explored.

Before the experiment started, each participant was shown both a set of target candies of all colors and a set of distractors of all colors to ensure they were able to distinguish between the two. All participants were given 7 min to explore the lawn before they were instructed to perform three search tasks. The first was unrelated to color: the instruction was to find and report as many targets as possible in 7 min. A found target was indicated by activating a laser-pointer and directing it at the target, while also looking at it (called “report” hereafter). The second and third search task were to exclusively search for and report targets of one specific color: red in the second task and yellow in the third task, with the same duration of 7 min each. To avoid any confounds by walking speed, time observers allotted to actual search, etc., all analysis considers relative numbers (fractions) of fixated items.

### Participants

A group of seven participants with color vision deficiencies (CVD, six male, one female, age 34.5 ± 9.6, ranged from 21 to 45) were invited after a positive Farnsworth D15 Test at home and later assessed with a Farnsworth-Munsell 100 Hue (Farnsworth, 1943) test. The inclusion criterion was a pathological test score [above the 95% confidence interval for the respective age group, (Kinnear and Sahraie, 2002)]. This criterion subsumes anomalous trichromats as well as dichromats. Five participants were of deutan, two of protan type. The FM 100 Hue test error scores were recorded. A control group (CTRL) of seven participants was recruited with the same procedure (six male, one female, age 32.9 ± 7.2, ranged from 25 to 43), and had normal results in both the D15 and the subsequent Farnsworth-Munsell 100 Hue tests. All fourteen participants gave their informed written consent. The experiments were approved by the ethics committee of the University of Munich Hospital and conducted in accordance with the declaration of Helsinki. During the recording of one CVD participant (protan type), an equipment malfunction led to unusable data. This participant was excluded from analysis, thus reducing the number of analyzed CVD participants to 6 (5 deutan, 1 protan). Three participants (2 CVD of deutan type, 1 CTRL) did not perform the condition “search for yellow targets” due to technical reasons, reducing the number of evaluable participants in this condition to 6 CTRL and 4 CVD.

### Data acquisition

During the experiments, the participants wore a mobile eye tracker with the unique ability to record a high resolution video of the wearer's retinal content (EyeSeeCam, Brandt et al., 2006; Schneider et al., 2009, Figure 1). The binocular eye tracking goggles are equipped with a head-fixed scene camera, and an additional moveable “gaze-camera” (see Supplementary Videos 1–3). The eye tracking data was binocularly evaluated in real time to continuously align the moveable camera with the determined gaze direction with a typical accuracy of 0.5° (Schneider et al., 2005) and a latency of ~13 ms (Schneider et al., 2009). Thus, the properties of biological retinal image stabilization mechanisms, i.e., vestibular ocular and optokinetic reflexes as well as smooth pursuit (Brandt, 2003), are all transferred to and used by the gaze camera. This way, a high-resolution video of the actual retinal content can be recorded, which has minimal motion-blur during fixations even under free body and head movements. This type of recording is a prerequisite for later analysis of gaze targets with image processing algorithms. The eye-tracking cameras operated at a frame rate of 304 Hz, while the scene camera and the gaze-aligned camera were synchronized and had a frame rate of 25 Hz.

Before the start of the experiment, the eye-tracker was calibrated. For calibration, a laser diode was mounted onto the motion platform of the gaze camera, thereby projecting a luminous point that appeared in the center of the gaze camera image (Schneider et al., 2005). During the calibration procedure the laser diode was switched on and participants were instructed to fixate the laser dot on the floor in front of them while it moved to 25 different positions. The calibration positions were chosen such that they optimally covered the field of view that was to be expected in the following tasks. The 25 recorded eye positions were used to fit a function that mapped measured eye positions to gaze camera positions. Before the start of the search tasks and subsequently every 2 min, participants were instructed to fixate an object in order to allow for manual correction of a possible goggle slippage. The participants carried the recording laptop and a laser pointer for reporting found targets by briefly pointing at them. Presses of the button on the laser pointer were recorded in synchrony with the gaze and scene movies as well as with the eye movement data.

### Data analysis

The eye-movement data was analyzed offline by a velocity-based algorithm with additional acceleration criteria (Ladda et al., 2007) in order to identify fixations and slow phases as well as saccades. Due to the unrestrained head and body movements, slow phase movements (optokinetic, smooth pursuit, and vestibulo-ocular reflex) were frequent. As their primary function is to stabilize gaze on a foveated target, all types of gaze-stabilizing eye-movements were, for simplicity, treated the same as “fixations.” We attributed one frame of the gaze-aligned video to every fixation calculated from the eye-tracking data. This frame was selected automatically to be the second frame captured after the start of the fixation. The selected frames were submitted to a custom-made object detection algorithm that determined present objects and their color. Afterwards, the detection results were verified manually. A circular region with a diameter of 2° in the video stream represented the foveal region of the retina. If one object was located in this region, we attributed the corresponding fixation to the specific color. When multiple or no objects were in the foveal region, the fixation was discarded. When the participants activated the laser pointer to report a found target, the corresponding recording time was excluded from analysis until the participant resumed the search. For each task, all fixations per objects of each color were summed up and normalized to the total number of evaluated fixations, resulting in a frequency distribution of fixated objects of the respective colors.

All recordings were manually evaluated for search performance, i.e., the number of correctly located targets. The number of search errors was determined for the task “search red” with an error categorization as follows: type I errors (false positive), i.e., a reported target was of correct shape but of wrong color, and type II errors (false negative), i.e., a correct target was fixated, but not reported. Both types of errors are made on an already fixated item, and thus relate to the final “verification” stage of search (Malcolm and Henderson, 2009), rather than to search *per-se*.

Statistical analysis was performed with MATLAB 2010a, Mathworks Inc. and R version 3.0.2. (R Core Team and R Foundation for Statistical Computing, 2013). Confidence intervals presented in figures were computed in MATLAB by applying the bias-corrected percentile bootstrap method.

## Results

### Search performance

**Figure 3:**
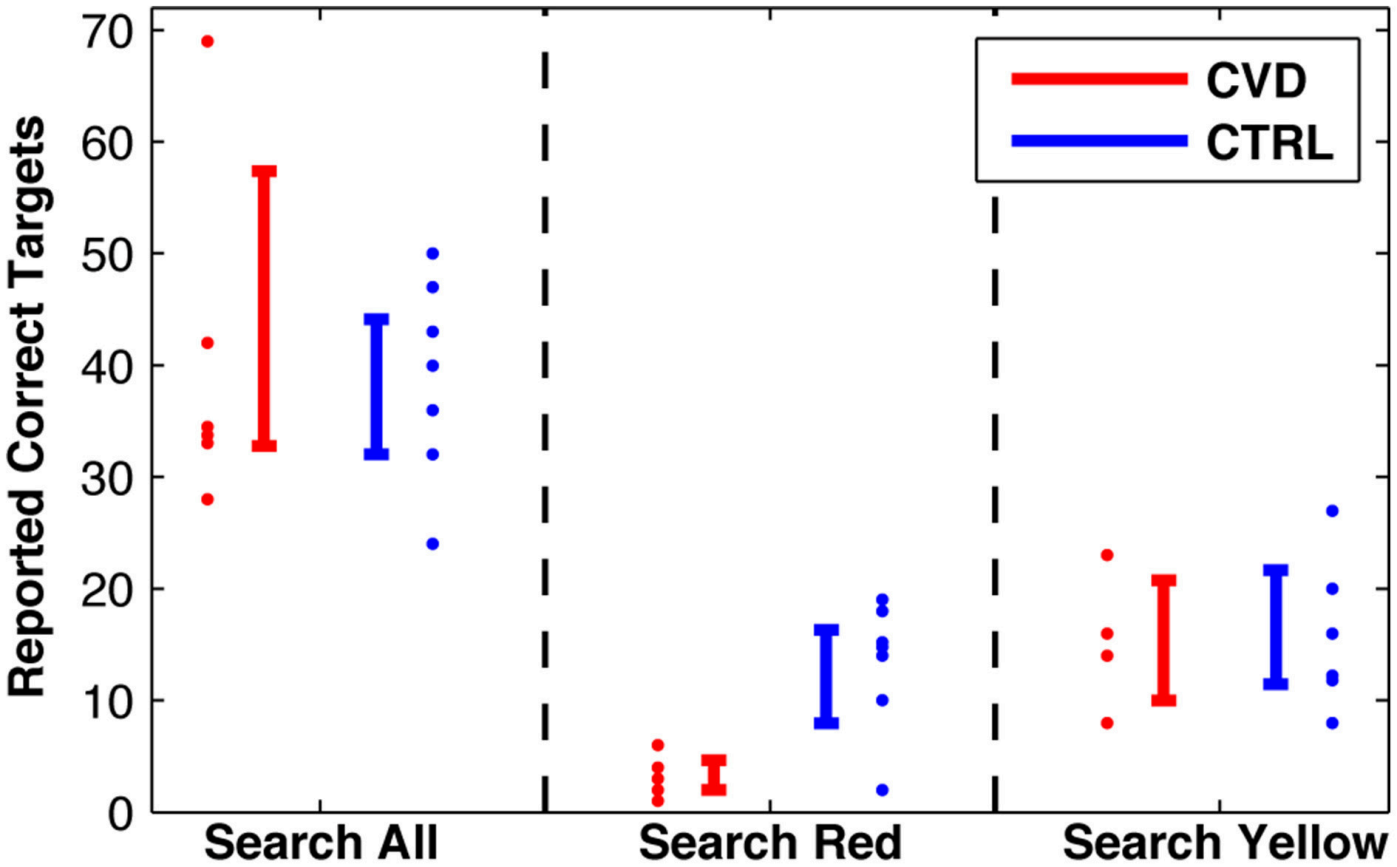
**Search performance, i.e., number of correct reported targets in the three conditions**. Individual data with 95%-confidence intervals.

As expected, the search performance of CVD participants was affected in the task “search red.” The correctly reported targets significantly differed between the CVD group (mean: 3.2) and the CTRL group [mean: 13.3, *t*_(11)_ = 4.12; *p* = 0.0017, Figure 3]. This difference still held when computed only for the participants who also performed the “search yellow” task [*t*_(8)_ = 2.89, *p* = 0.020]. In the task “search all,” in contrast, there was no difference of the mean number of correctly reported targets for the CVD group (39.8) and the CTRL group [38.8; *t*_(11)_ = 0.145, *p* = 0.89]. Similarly, in “search yellow,” there was also no difference between the CVD group (15.3) and the CTRL group [15.8; *t*_(8)_ = 0.137, *p* = 0.89].

An evaluation of verification errors for the condition “search red” revealed that CTRL did not exhibit any errors when identifying the target color. Three of six CVD reported candies of correct shape, but wrong color (type I error, seven in total, three brown, three orange, one green), leading to a mean value of 1.2 type I errors per participant. A mean value of 0.5 type II errors per participant (fixated but not reported correct targets) was made by the CVD group.

### Fixations

**Figure 4:**
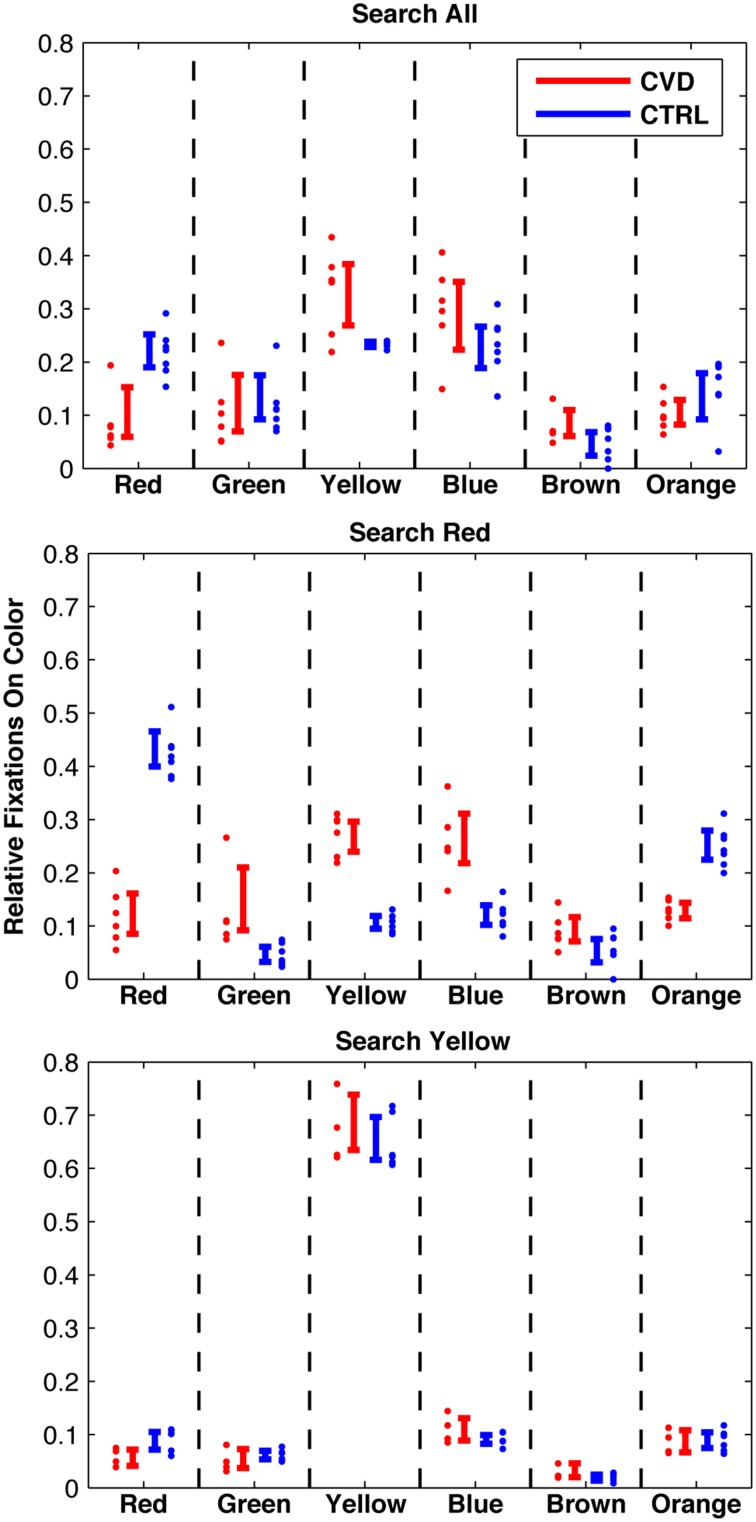
**Fixations per color in three conditions: Search targets of any color (top); search red targets (middle); search yellow targets (bottom)**. Individual data and 95%-confidence intervals are shown. Note: Significant difference between the groups in “search red” condition, while the two groups are remarkably similar in the “search yellow” condition. Also note that the CVD group exhibits a similar pattern in “search all” and “search red.”

Typically, about 500 fixations on single objects (mean and standard deviation 505 ± 127) were evaluated per participant and condition in order to calculate the proportions of fixations on objects of the six colors red, green, yellow, blue, brown, and orange. The proportion of fixations on the respective colored objects (short: “fixations on colors”) are shown in Figure 4 for all three conditions and both groups.

Differences of proportions of fixations on a specific color between the two groups were tested with two *t*-tests after arcsine transformation. The proportion of fixations on yellow objects in the search for yellow targets did not differ between the CVD and the CTRL groups [67 and 65%, respectively; *t*_(8)_ = 0.63, *p* = 0.55]. In contrast, the proportion of fixations on red objects in the search for red targets significantly differed between the CVD and the CTRL groups [11.9 and 42.4%, respectively; *t*_(11)_ = 11.0, *p* < 0.001]. This difference still held when only including those participants who also contributed to the search for yellow condition [*t*_(8)_ = 11.0, *p* < 0.001]. For comparison, CVD participants showed similar proportions of fixations on red objects in both the search for red targets (11.9%) and in the color-irrelevant search task [8.6%; *t*_(10)_ = 1.06, *p* = 0.31].

The Kendall-rank correlation of the test score of the Farnsworth-Munsell 100 Hue Test and the proportion of fixated red objects reveals a trend (τ = −0.733, *p* = 0.056).

## Discussion

We compared the performance of participants with color vision deficiencies with controls with normal color vision in three search tasks in natural conditions. We found no differences in search performance between the two groups in the search for yellow targets or when the target color was irrelevant. In the search for red targets, however, those with color vision deficiencies did show a significantly worse performance than controls, by reporting only 24% of the number of correct targets that controls reported.

We were especially interested in the question of whether gaze behavior could provide an explanation for the performance results. In the search for yellow targets, the CVD group exhibited a similar gaze behavior as the control group. In both groups, two out of three fixated objects were yellow. Consequently, both groups exhibited similar results in fixational data as well as in search performance. Thus, in this task, the remaining chromatic and luminance contrasts available for observers with color vision deficiencies suffice to select possible relevant items, i.e., yellow items, in the periphery for foveal inspection at a similar level as controls. In contrast, in the search for red targets, the proportion of fixated red objects differed between the groups. In particular, the participants with color vision deficiencies were not able to substantially increase their proportion of fixated red objects in the search for red targets (12%) as compared to the color-irrelevant search task (9%). Thus, the participants with color vision deficiencies are not able to use the residual chromatic and luminance contrasts of the red objects to guide gaze to red targets to the same extent as participants with normal color vision. In principle, based on the behavioral and eye-tracking data alone, two different mechanisms could underlie the altered results exhibited by the participants with color vision deficiency: confusion of red objects with objects of other colors, or insufficient residual contrast between the red objects and the background. In the first case, we would expect that the task “search red” would lead to increased fixations on colors that are confused with red together with increased fixations on red. Our results show that there is little to no increase in fixations on red nor observable changes in fixations on other colors. This provides evidence against the notion that our findings result from a confusion of red objects with distractors of other colors. The observed upper threshold of fixations on red matches the second interpretation, suggesting that the remaining contrast of red objects on the background is critically low, thus limiting the amount of peripherally detected red objects. This is confirmed by the chromaticities of the objects and the background (Figures 2B,C), which indicate that for participants with color vision deficiencies red objects are similar to the background lawn.

The proportion of fixated red objects is as low as one quarter of that of participants with normal color vision, closely resembling the reduced number of reported correct targets. This finding indicates that the ability to select objects of the relevant color in the periphery has strong influence on the search performance of people with color vision deficiencies. An interesting finding was that type II errors (i.e., correct red targets that were fixated, but not reported) rarely occurred. Once a red target is fixated, participants with color vision deficiencies are mostly able to identify it correctly. This further supports the notion that the main influence on search performance is indeed the degraded ability to select objects of the relevant color in the periphery of the visual field for subsequent fixation and foveal inspection. The identification is not as accurate as that of controls, as some errors (type I, i.e., target of wrong color is reported) occur. Thus, errors in our study were far less frequent than correct reports and half of the participants with color vision deficiencies did not make any error in identifying a foveated item as target. Taken together, search impairments in participants with color vision deficiencies result mainly from peripheral guidance, rather than from foveal discrimination between distractor and target color.

A surprisingly large fraction of people with color vision deficiencies are not aware of their altered color vision, especially when the alteration is mild. These numbers are as high as 25% of anomalous trichromats and 5% of dichromats (Steward and Cole, 1989). Our results show that participants with color vision deficiencies are able to find and identify red targets. This explains that the subjective experience for people with color vision deficiencies might be indistinguishable from “normal” experience, which is supported by the finding that the basic concept of naming colors in terms of red, green, yellow and blue is similar in dichromats (Wachtler et al., 2004). Still the difference in tasks in which peripheral selection is involved might be substantial even in the mild forms of color vision deficiency.

The variability in the proportion of fixated red objects is higher in the CVD group, as can be expected by the heterogeneous color vision abilities of the participants. Nevertheless, the participant with the highest rate of fixated red objects (and the least severe color vision deficiency in our group) is well below the lowest color normal (20 vs. 38%). Even with the small number of participants, the correlation of fixated red objects with classical error score in the FM 100 Hue test reveals a trend, suggesting that the ability to peripherally select red objects is dependent on the severity of the color vision deficiency.

The results on search performance match previous reports in the literature. O'Brien et al. (2002) found that yellow and blue coded signs could be detected by deuteranopes as well as by color-normals. In a search for clumps of red berries in a photograph, participants with color vision deficiencies performed worse than color-normal controls (Cole and Lian, 2006). Accordingly, in a search for a diamond-shaped target amongst differently shaped distractors (Cole et al., 2004), participants with color vision deficiencies performed worse than controls, especially when green and yellow color coding was used, suggesting that the participants with color vision deficiencies had difficulties in discriminating yellow and green to some extent. This is explained by the specific yellow and green used in that study; those colors were placed close to the dichromatic confusion axes in color space.

In the two tasks restricting the color of the target to one specific color, the controls fixated significantly more objects of the respective color as compared to the task in which color was irrelevant. This “guiding function” of color has been shown in numerous studies in the laboratory (Williams, 1967; Wolfe et al., 1989). The results of our study suggest that this search behavior transfers to a more natural setting. In contrast to the laboratory, the participants move the body and head continuously to access new parts of the lawn and the objects in it. Nevertheless, the gaze strategy remains similar to the previously examined laboratory conditions.

Selecting objects for foveal inspection is related to the ability to identify interesting locations in the peripheral visual field. The saliency map-model (Itti et al., 1998) proposes an internal “map” of the surroundings, where salient features (i.e., high luminance contrast, color contrast) are combined, and the most salient location is selected for foveal inspection. Primate trichromatic vision developed late during evolution around 30–40 million years ago (Jacobs, 2009). One major driving factor might have been the improved detection of ripe fruit in foliage for the frugivorous primates (Frey et al., 2011). Our study suggests that the specific color and luminance contrasts between the food and the foliage are of crucial importance for the detection of ripe fruit. Whether there is sufficient contrast to allow efficient peripheral selection, will strongly affect search performance.

## Conclusion

The reduced chromatic and luminance contrasts available to those with color vision deficiencies can lead to strongly impaired search performance. In our tasks, this impairment covered a wide range, from a behavior close to normal to strong impairment, which trends to be related to the severity of the color impairment. Importantly, peripheral guidance is strongly affected by the color-vision deficiency, whereas foveal identification is largely unimpaired.

### Conflict of interest statement

The authors declare that the research was conducted in the absence of any commercial or financial relationships that could be construed as a potential conflict of interest. Four of the authors, GK, SK, KB, and ES are shareholders of EyeSeeTec GmbH, owner of the trademark EyeSeeCam.

## Supplementary material

Data file accompanying this report is available online at the figshare repository (Kugler et al., 2015).

Supplementary Video 1 **Participant during search in lawn**.

Supplementary Video 2 **Combined recording of head-fixed scene shot and gaze-directed camera**. The black circle in the head-fixed shot shows the current direction of gaze. Note: Candies in head-fixed short are too small to be evaluated, while the gaze-camera delivers magnified shots that can be evaluated by an object-detection algorithm.

Supplementary Video 3 **Recorded gaze-aligned video during search for targets of any color**. The video frames have been analyzed by an object-detection algorithm. All detected objects have been marked in the video. The circle represents the region of the frames corresponding to the fovea. Note: The participant is fixating candies repeatedly, as can be seen when following the inside of the circular region.
